# NEIL3 influences adult neurogenesis and behavioral pattern separation via WNT signaling

**DOI:** 10.1007/s00018-025-05629-5

**Published:** 2025-03-04

**Authors:** Marion S. Fernandez-Berrocal, Amilcar Reis, Veslemøy Rolseth, Rajikala Suganthan, Anna Kuśnierczyk, Arthur França, Annara Y. M. Soares, Nicolas Kunath, Anna M. Bugaj, Andreas Abentung, Lars Eide, Richardson N. Leão, Magnar Bjørås, Katja Scheffler, Jing Ye

**Affiliations:** 1 Department of Clinical and Molecular Medicine (IKOM), Norwegian University of Science and Technology (NTNU), 7491 Trondheim, Norway; 2 Proteomics and Metabolomics Core Facility, PROMEC, Norwegian University of Science and Technology (NTNU), 7491 Trondheim, Norway; 3 Department of Neuromedicine and Movement Science, Norwegian University of Science and Technology (NTNU), 7491 Trondheim, Norway; 4 Department of Neurology and Clinical Neurophysiology, University Hospital of Trondheim, 7030 Trondheim, Norway; 5 Department of Microbiology, Oslo University Hospital, University of Oslo, 0424 Oslo, Norway; 6 Centre for Embryology and Healthy Development, University of Oslo, 0373 Oslo, Norway; 7 Department of Neuroscience, Uppsala University, 752 36 Uppsala, Sweden; 8 Neurodynamics Lab, Brain Institute, Federal University of Rio Grande Do Norte, Natal, 59056-450 Brazil; 9 Department of Medical Biochemistry, University of Oslo, Oslo, Norway

**Keywords:** NEIL3 DNA glycosylase, Oxidative DNA damage, Neural stem and progenitor cells (NSPCs), Novel object location (NOL), Hippocampal transcriptome, Patch-clamp recording

## Abstract

Adult neurogenesis in the hippocampus, involving the generation and integration of new neurons, is essential for behavioral pattern separation, which supports accurate memory recall and cognitive plasticity. Here, we explore the role of the DNA repair protein NEIL3 in adult hippocampal neurogenesis and behavioral pattern separation. NEIL3 is required for efficient proliferation and neuronal differentiation of neonatal NSPCs and adult-born NPCs in the hippocampus following a behavioral pattern separation task. NEIL3-depleted mice exhibited a reduced preference for the novel object location, indicating a deficit in pattern separation. NEIL3-deficient adult-born neurons exhibited a significant reduction in mature-like membrane properties, indicating impaired functional maturation. Interestingly, these impairments were not associated with the decreased genomic integrity but with the altered transcriptional regulation of the Wnt signaling pathway. Given the importance of adult neurogenesis in cognitive function, targeting NEIL3 could offer therapeutic potential for addressing age-related hippocampal dysfunction and cognitive decline.

**Supplementary Information:**

The online version contains supplementary material available at 10.1007/s00018-025-05629-5.

## Introduction

Adult hippocampal neurogenesis in the mammalian brain generates new neurons in the subgranular zone (SGZ) of the dentate gyrus (DG) throughout life. The maturation of dentate granule cells (DGCs) occurs in a complex and multistep process. Slowly dividing radial glia-like type 1 neuronal stem cells (NSCs) give rise to more rapidly dividing type 2 neuronal progenitor cells (NPCs) [1–4]. Molecular markers like SOX2 are associated with type 2 NPCs, while NeuroD1 and DCX are indicative of type 3 NPCs [5, 6]. After commitment to the neuronal lineage adult-born DGCs travel a short distance to the granule cell layer (GCL) and undergo a lengthy process of morphological and physiological maturation before they functionally integrate into the DG circuitry [7, 8]. Newly generated DG neurons contribute to pattern separation, a process crucial for distinguishing similar yet distinct memories, allowing for accurate memory recall and improved cognitive plasticity [9–12].

The process of adult neurogenesis is known to generate localized oxidative stress by reactive oxygen species (ROS) [13, 14]. Although the effects of ROS in adult neurogenesis are not well studied, their accumulation has been linked to various clinical neuropathologies, including Alzheimer’s disease[15] and amyotrophic lateral sclerosis [16]. Oxidized DNA base lesions are mainly processed by base excision repair (BER), which is initiated by DNA glycosylases [17]. NEIL3 is one of several mammalian DNA glycosylases that recognize and excise damaged bases in particular oxidation products of 8-oxoguanine (8-oxoG), spiroiminodihydantoin (Sp), and guanodinohydantoin (Gh) from single-stranded DNA [18]. The expression pattern of NEIL3 in the rodent brain is unique among DNA glycosylases, being specifically localized to highly proliferative cells in the subventricular zone (SVZ) and DG-SGZ [19, 20]. NEIL3 expression is notably higher during early postnatal development compared to adulthood [21, 22]. Our previous research has highlighted the important role of NEIL3 in various processes, including neural progenitor cell growth [23], post-stroke neurogenesis [21], hippocampal maturation [22, 24], neuronal plasticity [22], and the hippocampus-dependent learning and memory [23, 25].

In the present study, we investigated the role of NEIL3 in adult hippocampal neurogenesis and its impact on hippocampal-dependent behavioral pattern separation.

## Results

### Loss of NEIL3 impairs both proliferation and neuronal differentiation of neonatal NSPCs

**Fig. 1:**
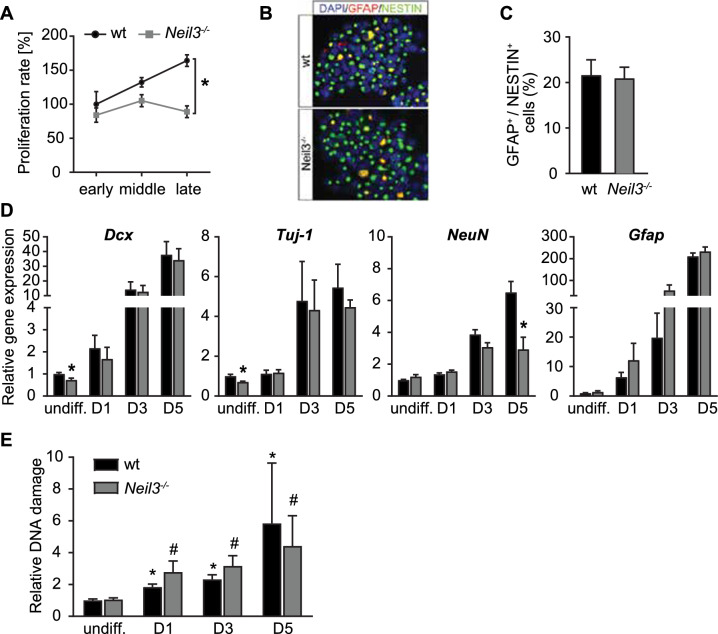
NEIL3 depletion impairs both proliferation and neuronal differentiation of neonatal NSPCs. A-C NSPCs were cultured from postnatal day 5 mouse hippocampi across genotypes (wildtype and *Neil3*^*−/−*^). (**A**) Percentage of proliferation rate calculated from cell counts at passages 2–5 (early), 6–10 (middle), 11–17 (late). (**B**) Representative images of GFAP and NESTIN staining in undifferentiated NSPCs. (**C**) Quantification of neural stem cells represented as percentages of GFAP- and NESTIN- double positive cells. D-F Undifferentiated (undiff.) and differentiated NSPCs (passages 5-11) for one day (D1), three days (D3) and five days (D5) across genotypes (wildtype and *Neil3*^*−/−*^). (**D**) Gene expression analysis of neuronal and glia markers across days of differentiation. (**E**) Gene expression of relative DNA damage level across days of differentiation. Statistical analyses were done in GraphPad Prism Software v10.1 using 2-way ANOVA with Tukey’s multiple comparisons test. All data are presented as mean ± SEM (n = 3), *p < 0.05 compared to wildtype, ^§^p < 0.05 compared to wildtype undifferentiated, ^#^p < 0.05 compared to *Neil3*^*−/−*^

Previous work with 18-month-old *Neil3*^*−/−*^ mice revealed impaired in vitro expansion of adult hippocampal neurospheres [23]. Here, we assessed neurosphere formation from neural stem and progenitor cells (NSPCs) derived from hippocampal tissue of postnatal-day-5 (p5) wildtype and *Neil3*^*−/−*^ animals. Primary neurosphere formation was successful for both genotypes with no significant differences in growth rate at early passages (passages 2-5). Continuous passaging increased the proliferation rate of wildtype but not *Neil3*^*−/−*^ neurospheres resulting in a 50% lower number of proliferating NSPCs at late passages (passages 11-17) (Fig. 1A). We could not detect differences in the number of slowly dividing GFAP^+^ NESTIN^+^ radial glia cells between the genotypes (Fig. 1B, C). However, gene expression of neuronal progenitor marker Dcx and early neuronal marker Tuj-1 was significantly decreased in proliferating NSPCs from later passages of *Neil3*^*−/−*^ neurospheres (Fig. 1D), while no such decrease was observed in early passages (Supplementary Figure S1A). To evaluate the differentiation potential of NSPCs from wildtype and *Neil3*^*−/−*^ hippocampus, we withdrew the epidermal growth factor and examined the gene expression of neuronal markers (Dcx, Tuj1, NeuN) and glia cell lineage markers (Gfap). All markers showed increased expression from the proliferative state to day 5 of differentiation in both wildtype and *Neil3*^*−/−*^ cells, indicating effective differentiation capacity (Fig. 1D). The highest NEIL3 expression was detected in proliferating NSPCs with a striking reduction in differentiated cells (Supplementary Figure S1B). Notably, a significant decrease in NeuN expression was observed in *Neil3*^*−/−*^ cells, indicating further impairment in the state of neuronal maturation. This finding aligns with our previous research, which highlighted the role of NEIL3 in hippocampal maturation through its influence on transcription[24]. There were no differences between wildtype and *Neil3*^*−/−*^ neurospheres to produce astrocytes (Fig. 1D). Next, we analyzed nuclear DNA damage levels in proliferating and differentiating neurospheres by real-time quantitative PCR. This method is based on the inability of a restrictive enzyme to cleave damaged DNA, as described in Wang 2016 [26]. Surprisingly, despite a significant accumulation of DNA damage during differentiation, the levels in *Neil3*^*−/−*^ cells were indistinguishable from those in wild-type cells (Fig. 1E).

### NEIL3 depletion impairs behavioral pattern separation and behavior-induced adult neurogenesis

**Fig. 2:**
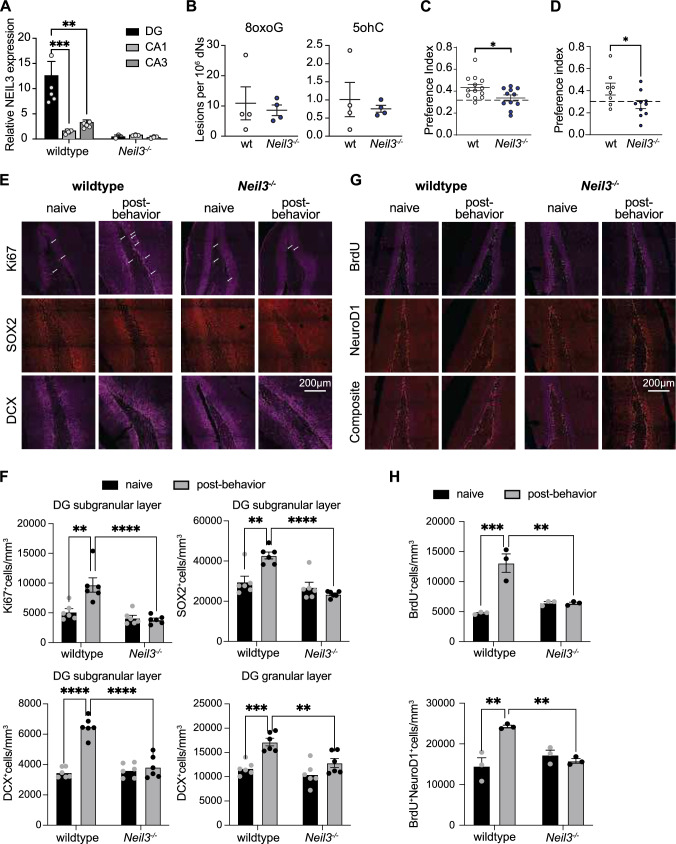
NEIL3 depletion impairs behavior pattern separation in mice and behavior induced adult neurogenesis. (**A**) The histogram shows the relative NEIL3 expression in hippocampal subregions of wildtype (n = 5) and *Neil3*^*−/−*^ (n = 3) mice quantified by RT-qPCR. (**B**) LC–MS/MS quantification of 8-oxoguanine (8-oxoG) and 5-hydroxycytosine (5ohC) in the hippocampus of wildtype and *Neil3*^*−/−*^ mice. Data are presented as mean ± SEM (n = 4), *p < 0.05. (**C-D**) The preference index for wildtype and *Neil3*^*−/*−^ animals reflects the time spent exploring the novel-located objects compared to a reference index (0.33, no preference for any object, dash line) in the short-term (C) and long-term (D) memory paradigms for pattern separation. Statistical analyses were done in GraphPad Prism Software v10.1 using t-test. Data are presented as mean ± SEM from wildtype (n = 13 mice) and *Neil3*^*−/−*^ (n = 11 mice) for short-term and wildtype (n = 7 mice) and *Neil3*^*−/−*^ (n = 12 mice) for long-term tests with *p < 0.05 and **p < 0.001. (**E**) Representative images show Ki67^+^ proliferating cells (magenta), SOX2^+^ neuro-progenitor cells (red), and DCX^+^ immature neurons (magenta) in the hippocampal-DG of wildtype and *Neil3*^*−/−*^ mice (naive vs post-behavior). The images display a maximal projection of five Z-stacks (1 µm each). All sections were co-stained with NeuN and DAPI (not shown). (**F**) Histograms show quantification of Ki67, DCX, and SOX2 positive cells per 1 m^3^ tissue volume in the DG-subgranular layer. Additionally, the number of DCX^+^ migrating cells in the DG-granular layer is also analyzed. (**G**) Representative images show BrdU^+^ (magenta) and NeuroD1^+^ (red) cells in the hippocampal-DG of wildtype and *Neil3*^*−/−*^ mice (naive vs post-behavior). The images display a maximal projection of five Z-stacks (1 µm each). All sections were co-stained with NeuN and DAPI (not shown). (**H**) Histograms show quantification of BrdU-positive and BrdU/NeuroD1 double-positive cells per 1 m^3^ tissue volume. The post-behavior animals are those passed the long-term pattern separation behavioral test. All data are presented as mean ± SEM (n = 6 mice), *p < 0.05, **p < 0.001, ***p < 0.0001, ****p < 0.00001

In the adult hippocampus, NEIL3 displayed the highest gene expression levels in DG, with significantly lower levels in CA1 and CA3 (Fig. 2A). Again, no accumulated oxidative DNA base lesions were detected in the adult hippocampus of *Neil3*^*−/−*^ mice, as measured by the levels of 8-oxoguanine (8-oxoG) and 5-oh cytosine (5-ohC) using liquid chromatography-tandem mass spectrometry (LC–MS/MS) (Fig. 2B). This suggests that NEIL3 is not crucial for the overall removal of oxidized DNA base lesions in the hippocampus.

Adult neurogenesis is a hallmark of DG, known for its critical role in cognitive functions, particularly in behavioral pattern separation in memory. We refined the Novel Object Location (NOL) task to assess both short-term and long-term memory in the context of behavioral pattern separation (see methods). In the short-term memory paradigm (1 training session and 1 test session with intertrial intervals of 5 min), wildtype animals spent significantly more time in the novel area than *Neil3*^*−/−*^ mice (mean values: 0.45 vs. 0.3, Fig. 2C). *Neil3*^*−/−*^ mice showed no preference for the novel location, spending similar time as indicated by the reference index. In the long-term memory paradigm (3 training sessions and 1 test session with a 24-h intertrial interval), wild-type animals also spent significantly more time exploring the novel area compared to *Neil3*^*−/−*^ mice (mean values: 0.48 vs. 0.28, Fig. 2D). Next, we evaluated adult neurogenesis in the hippocampal DG subgranular layer of wildtype and *Neil3*^*−/−*^ animals (naive vs post-behavior) using immunohistochemistry with various neural proliferation and differentiation markers. Increased numbers of Ki67^+^ proliferating cells, SOX2^+^ neuroprogenitors, and DCX^+^ immature neurons were observed in wildtype mice following the long-term memory paradigm for behavioral pattern separation, but not in *Neil3*^*−/−*^ mice (Fig. 2E, F), suggesting impaired adult neurogenesis in the NEIL3-deficient DG. Notably, significantly decreased DCX^+^ migrating cells were detected in the DG granular layer in mice lacking NEIL3 (Fig. 2F). Additionally, we tracked newborn neurons in the DG by administering BrdU intraperitoneally to wildtype and *Neil3*^*−/−*^ mice prior to the behavioral task (see methods). BrdU-labeled newborn neurons were visualized through co-staining with BrdU and NeuroD1. Again, a significant increase in BrdU-labeled newborn neurons was observed in wildtype mice after the long-term memory paradigm for behavioral pattern separation, but this increase was absent in *Neil3*^*−/−*^ mice (Fig. 2G, H). Taken together, these results suggest that NEIL3 depletion impairs behavioral pattern separation and behavior-induced hippocampal adult neurogenesis.

### NEIL3 impacts hippocampal adult neurogenesis by regulating the WNT signaling pathway

**Fig. 3:**
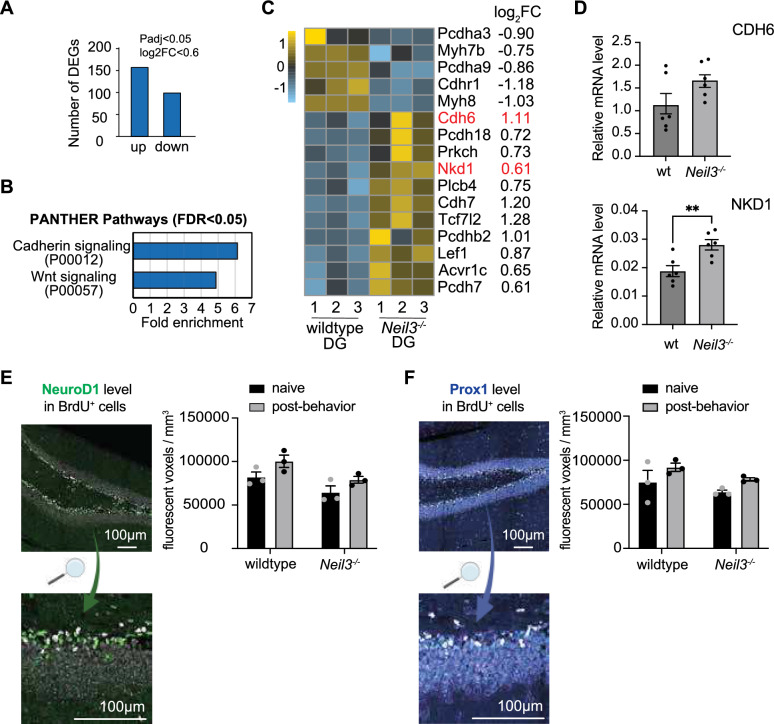
NEIL3 impacts hippocampal adult neurogenesis by regulating the WNT signaling pathway. (**A**) The number of up- and down-regulated genes in *Neil3*^−/−^ DG (p_adj_ < 0.05, n = 4 for wildtype and n = 4 for *Neil3*^*−/−*^ mice). (**B**) PANTHER pathway analysis of NEIL3-associated DEGs revealed overrepresented Cadherin and Wnt signaling pathways (P00012 and P00057). False Discovery Rate (FDR) < 0.05. (**C**) The heatmap illustrates DEGs involved in the Cadherin and Wnt signaling pathways. It shows z-score normalized FPKM expression levels for the selected genes across genotypes (wildtype and *Neil3*^*−/−*^). The log2 fold change (log2FC) values of the respective DEGs are indicated. (**D**) The relative mRNA levels of Cadherin 6 (CDH6) and NKD Inhibitor of WNT Signaling Pathway 1 (NKD1) in wildtype and *Neil3*^*−/−*^ DG were shown. Data is presented as mean ± SEM (n = 6 mice), **p < 0.001 (**E**) A representative image shows the NeuroD1^+^ (green), BrdU^+^ (magenta), and the double-positive (white) cells in the hippocampal DG. The numbers of NeuroD1^+^ fluorescent voxels within BrdU^+^ cells in DG of wildtype and *Neil3*^*−/−*^ mice (naive vs post-behavior) were quantified. (**F**) A representative image shows the Prox1^+^ (blue), BrdU^+^ (magenta), and the double-positive (white) cells in the hippocampal DG. The numbers of Prox1^+^ fluorescent voxels within BrdU^+^ cells in DG of wildtype and *Neil3*^*−/−*^ mice (naive vs post-behavior) were quantified. The post-behavior animals are those passed the long-term pattern separation behavioral test. Data is presented as mean ± SEM (n = 3 mice), *p < 0.05

Our previous work has demonstrated the important role of NEIL3 in hippocampal maturation and function by influencing transcription [22, 24]. To further explore the NEIL3-associated molecular mechanisms in adult neurogenesis, we performed a specialized analysis of the DG transcriptome from adult wild-type and *Neil3*^*−/−*^ mice, utilizing sequencing data published in Bugaj et al., 2024 [24]. In the *Neil3*^*−/−*^ DG, 256 differentially expressed genes (DEGs) were identified (Log_2_FC > 0.6 and P_adj_ < 0.05), with 157 genes upregulated and 99 downregulated (Fig. 3A). PANTHER pathway analysis revealed that NEIL3-associated DEGs were significantly overrepresented in the Cadherin and Wnt signaling pathways (P00012 and P00057, FDR < 0.05) (Fig. 3B). Genes involved in the Wnt signaling pathway are displayed in the heat plot (Fig. 3C). Additionally, β-catenin (Ctnnb1) was mildly upregulated (log2FC = 0.22, Padj = 0.002) in *Neil3*^*−/−*^ DG. The upregulation of Cadherin 6 (CDH6) and NKD inhibitor of Wnt signaling pathway 1 (NKD1) was further validated by RT-qPCR (Fig. 3D). Wnt signaling is known to regulate neuronal differentiation through the proneural transcription factors including Ngn2, NeuroD1, and Prox1[27]. We analyzed the level of NeuroD1 and Prox1 by measuring fluorescent voxels in BrdU-labeled newborn neurons and found consistently decreased levels of both NeuroD1 and Prox1 in *Neil3*^*−/−*^ animals, in naive and post-behavior conditions (Fig. 3E, F). These results suggest that NEIL3 impacts hippocampal adult neurogenesis by regulating the WNT signaling pathway.

### NEIL3 depletion impairs the functional maturation of newborn DG neurons

**Fig. 4:**
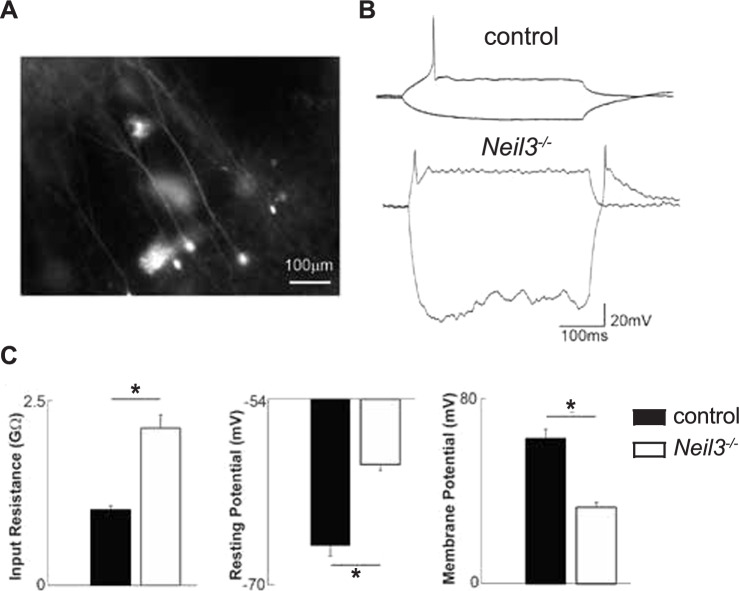
NEIL3 depletion impairs the functional maturation of adult-born hippocampal neurons. Patch clamp recordings from DG neurons in *Neil3*^+/-^ ::DCX-CreER (control) and *Neil3*^-/-^::DCX-CreER mice. (**A**) Example of recorded and filled neurons in the dentate gyrus. (**B**) Representative current-clamp recordings showing firing properties of DG neurons. (**C**) Combined quantitative data showing input resistance, resting potential and membrane potential across genotypes (*Neil3*^+/-^ and *Neil3*^*−/−*^). Statistical analyses were done in GraphPad Prism Software v10.1 using t-test. Data are presented as mean ± SEM (n = 18 cells), *p < 0.05

Lastly, we assessed the physiological characteristics of newborn DG neurons in *Neil3*^*−/−*^::DCX-CreER mice using patch-clamp recordings. To label these neurons, we performed viral transduction of a fluorescent reporter, followed by tamoxifen injection (see methods). Newborn neurons were identified by eYFP expression under epifluorescence (Fig. 4A). We recorded eYFP^+^ newborn cells in *Neil3*^+/-^::DCX-CreER2 (control, n = 9 mice) and *Neil3*^*−/−*^::DCX-CreER2 mice (n = 9 mice). The *Neil3*^*−/−*^ cells exhibited membrane properties compatible with immature granule cells (GCs) (Fig. 4B). Notably, their input resistance was significantly higher (2.13 ± 0.18 GΩ,) compared to *Neil3*^+/-^ control cells (1.02 ± 0.05 GΩ) (n = 18 cells, p = 0.00002, *t*-test, Fig. 4C). Additionally, *Neil3*^*−/−*^ newborn neuron displayed a significantly more depolarized resting membrane potential (− 59.68 ± 0.50 mV) compared to *Neil3*^+/-^ control cells (− 66.70 ± 0.91 mV, n = 18 cells, p = 0.000004, t-test) and a reduced action potential amplitude (33.11 ± 2.02 mV in *Neil3*^*−/−*^ vs 62.89 ± 3.96 mV in *Neil3*^+/-^, n = 18 cells, p = 0.000005, t-test, Fig. 4C). Moreover, we recorded mature DG granule cells in *Neil3*^*−/−*^::DCX-CreER (n = 7 mice) and *Neil3*^+/-^::DCX-CreER (n = 5 mice) mice, finding no significant differences in input resistance (409 ± 36.6 MOhm vs. 370 ± 31.8 MOhm, respectively, n = 24 cells, Supplementary Figure S2A), resting membrane potential (− 73.3 ± 2.6 mV in *Neil3*^+/-^ vs 73.6 ± 3.0 mV in *Neil3*^*−/−*^, n = 24 cells), action potential (AP) amplitude (80.7 ± 4.8 mV in *Neil3*^+/-^ vs. 79.8 ± 4.2 mV in *Neil3*^*−/−*^, n = 24 cells) and AP half-width (of the first AP elicited by current steps) (1.7 ± 0.19 ms in *Neil3*^+/-^ vs 1.3 ± 0.05 ms in *Neil3*^*−/−*^, n = 24 cells). However, *Neil3*^*−/−*^ cells showed a significantly reduced after-hyperpolarization potential (following the first AP: − 17.1 ± 3.4 mV in *Neil3*^+/-^ vs. − 8.5 ± 1.3 mV in *Neil3*^*−/−*^ cells, p = 0.04, n = 24 cells, Supplementary Figure S2B) and a greater sag response produced by − 100 pA hyperpolarizing step (− 0.38 ± 0.61 mV in *Neil3*^+/-^ vs. − 3.51 ± 0.54 mV in *Neil3*^*−/−*^ cells, p = 0.001, n = 24 cells, Supplementary Figure S2C). Further, we performed post hoc reconstruction and Sholl analysis [28] on biocytin-filled *Neil3*^+/-^ and *Neil3*^*−/−*^ granule cells (Supplementary Figure S2D), revealing significant differences in dendritic branching. At 100uM from the soma, *Neil3*^*−/−*^ cells had significantly fewer branches (3.63 ± 0.09) compared to *Neil3*^+/-^ cells (6.5 ± 0.09, N = 16, p < 0.01). Taken together, these findings suggest that *Neil3*^*−/−*^ newborn (DCX +) neurons take longer to develop mature-like membrane properties, while NEIL3-deficient mature neurons exhibit reduced dendritic branching, highlighting an important role of NEIL3 role in the functional maturation of adult-born DG neurons.

## Discussion

Unlike other DNA glycosylases, NEIL3 mRNA expression is restricted to neurogenic niches in the brain [19, 20]. As expected, we found the highest expression in DG of the adult hippocampus (Fig. 2A), corresponding to the brain region with proliferating stem cell populations. Loss of NEIL3 has previously been shown to impair in vitro expansion of adult NSPCs from the aged hippocampus [23] and to affect stroke-induced neurogenesis [21]. Here, we found that NSPCs from the *Neil3*^*−/−*^ neonatal hippocampus initially expand like wildtype cells but fail to increase their proliferation rate during propagation (Fig. 1A). In vivo, previous studies indicate that NEIL3 depletion did not affect the number of proliferating cells in the perinatal hippocampal DG in naive mice, as assessed by the proliferation marker Ki67 and BrdU incorporation [24, 29]. Interestingly, in a mouse model of Alzheimer’s disease, NEIL3 depletion impaired adult hippocampal neurogenesis and hippocampus-dependent working memory [25]. Notably, *Neil3*^*−/−*^ mice exhibited impaired hippocampus-dependent learning and memory as tested in the Morris water maze task and reduced anxiety-like behavior in the elevated zero maze [23]. This study revealed a significant reduction in proliferating cells (marked by Ki67 or BrdU), neuro-progenitors (marked by Sox2), and immature neurons (marked by DCX) in the DG of *Neil3*^*−/−*^ animals following a behavioral pattern separation task (Fig. 2E–H), indicating an important role of NEIL3 in the behavior-induced adult neurogenesis. Behavioral pattern separation is a cognitive process crucial for differentiating similar experiences and forming accurate, contextually appropriate memories [30]. This process depends on the integration and functional maturation of new neurons in the hippocampal circuitry [31]. Research has shown that disrupting hippocampal adult neurogenesis impairs spatial discrimination of stimuli with minimal separation [31]. Conversely, enhancing neurogenesis in the DG by increasing the survival of adult-born neurons improves pattern separation [32]. Consistent with these findings, *Neil3*^*−/−*^ mice exhibited impaired behavioral pattern separation (Fig. 2C–D), likely due to disrupted neurogenesis in the adult DG following various experiences. Furthermore, electrophysiological measurements of newborn DCX^+^ DG neurons revealed that NEIL3 depletion impairs their functional maturation (Fig. 4). This finding aligns with our earlier observations of delayed hippocampal maturation due to NEIL3 depletion [22, 24]. Thus, our study identifies NEIL3 as a positive regulator of neuronal maturation in the hippocampus.

The process of adult neurogenesis is known to generate localized oxidative stress by reactive oxygen species (ROS) [13, 14]. In the brain, oxidized DNA base lesions are mainly processed by base excision repair (BER), initiated by DNA glycosylases [17]. NEIL3 is the main glycosylase that removes the oxidation products of 8-oxoguanine (8-oxoG), including spiroiminodihydantoin (Sp) and guanodinohydantoin (Gh), as well as 5-Hydroxymethylcytosine (5-ohC) [18]. Notably, depletion of NEIL3 does not lead to a global accumulation of 8-oxoG and 5-ohC in the hippocampal genome (Fig. 2B), suggesting that NEIL3 is not essential for the overall removal of oxidized DNA base lesions during hippocampal adult neurogenesis. Our recent studies have highlighted the important role of NEIL3 in shaping the hippocampal transcriptome during development and behavior [22, 24]. Here, we identified differentially expressed genes (DEGs) in the *Neil3*^*−/−*^ DG that are involved in the Wnt signaling pathway (Fig. 3). The Wnt pathway is crucial for adult neurogenesis, influencing the proliferation, differentiation, and integration of new neurons in DG [27]. Notably, Wnt-target genes involved in neuronal differentiation and maturation, such as NeuroD1 [33] and Prox1 [34], exhibit reduced expression in behavior-induced newborn neurons in *Neil3*^*−/−*^ DG (Fig. 3E–F). Our findings suggest that NEIL3 modulates Wnt-mediated activation of behavior-induced adult neurogenesis. However, the molecular mechanisms by which NEIL3 regulates transcription require further investigation. It is tempting to speculate that NEIL3 may initiate BER on gene-specific DNA sequences to regulate gene expression. For example, NEIL3 may function in gene regulation by removing oxidized lesions in quadruplex structures of promoter regions [35].

## Conclusion

In summary, this study shows that NEIL3 regulates hippocampal adult neurogenesis and behavioral pattern separation through the WNT signaling pathway. Given the importance of adult neurogenesis in cognitive function, targeting NEIL3 could offer therapeutic potential for addressing age-related hippocampal dysfunction and cognitive decline.

## Materials and methods

### Animals

Mice used in this study were on C57BL6 background. *Neil3*^*−/−*^ animals were generated as previously described [36]. The DCX-CreERT2 mouse model (Stem Cell Research, Helmholtz Center Munich) is a genetically engineered lineage with CreERT2 expression specifically restricted to doublecortin (DCX) expressing neural progenitor cells. The *Neil3*^+/-^::DCX-CreER2 and *Neil3*^−/−^::DCX-CreER2 mouse models were generated by crossing *Neil3*^−/−^ and DCX-CreER2 lines, allowing targeted genetic manipulation of DCX-expressing neural progenitor cells in a NEIL3-deficient background. The *Neil3*^+/-^::DCX-CreER2 mice were used as controls in patch clamp recording experiments. For behavioral studies, 5-week-old mice were used, for other experimental procedures 3- to 6-month-old mice were used unless stated otherwise. The mice were bred and housed in 12-h light and dark cycle with food and water ad libitum. All experiments were approved by the Norwegian Animal Research Authority and conducted in accordance with laws and regulation controlling experimental procedures in live animals in Norway and the European Union Directive 86/609/EEC and experiments conducted in Brazil were approved by the Animal Ethics Committee of the Federal University of Rio Grande do Norte (CEUA Proj. No. 051/2015).

### Isolation and culture of NSPCs

Hippocampal NSPC were prepared from postnatal day 5 old mice and propagated as described previously [37]. Briefly, neurospheres were cultured for 7 days in a proliferation medium consisting of Neurobasal-A medium with 2% B27 supplement, 20 ng/ml basic fibroblast growth factor, 10 ng/ml epidermal growth factor, 2 mM L-glutamine, and penicillin/streptomycin. For analysis of the proliferation rate, single cells were counted at each passage using the Countess Automated Cell Counter (Invitrogen). For immunostaining of undifferentiated NSPCs, single cells were immobilized on a slide using a Thermo Shandon Cytospin (Thermo Fisher) centrifuge at 800 rpm for 4 min before fixation with 4% PFA. Cells were blocked with 5% BSA, 5% goat serum and 0.5% Triton-X-100 in PBS for 1 h followed by incubation with primary antibodies in PBS containing 0.5% BSA and 0.5% goat serum at 4 °C overnight. Staining with secondary antibodies was performed for 1 h at room temperature (RT). The percentage of double-positive cells was calculated in relation to the total number of cells visualized by DAPI nuclear staining (1 μg/ml, Invitrogen). For differentiation, single cells were plated onto poly-D-lysine-coated plates in proliferation medium without epidermal growth factor. Cultures from at least three different mice of each genotype were expanded separately.

### Extraction of nucleic acid

Genomic DNA from NSPC cultures was isolated using the DNeasy Blood and Tissue kit and total RNA using the RNeasy Kit (Qiagen) following the manufacturer’s protocol. DNA and RNA from mouse hippocampal tissue were extracted using the AllPrep DNA/RNA/Protein mini Kit (Qiagen). Briefly, tissue was homogenized in RLT lysis buffer using Fastprep^R^-24 instrument, centrifuged for 3 min, 13,000 rpm, and supernatant was used for nucleic acid isolation.

### DNA damage detection

Nuclear DNA damage was analyzed using a real-time qPCR-based method described previously [26]. Briefly, genomic DNA was digested with Taq^α^I restriction enzyme, and subsequent real-time PCR was carried out with 6 ng total DNA. Relative amounts of PCR products were calculated by the comparative ΔΔCT method. The following primers were used: forward: 5’-tggtgactcctacctgaagc and reverse, 5’-ttcggtcgtgaattttgtt.

### LC–MS/MS quantification

DNA samples were digested by incubation with a mixture of nuclease P1 from Penicillium citrinum (SIGMA, N8630), DNase I (Roche, 04716728001) and ALP from E. coli (SIGMA P5931) in 10 mM ammonium acetate buffer pH 5.3, 5 mM MgCl_2_ and 1 mM CaCl_2_ for 30 min at 40 °C. The samples were methanol precipitated, supernatants were vacuum centrifuged at room temperature until dry, and dissolved in 50 μl of water for LC/MS/MS analysis. Quantification was performed with the use of an LC-20AD HPLC system (Shimadzu) coupled to an API 5000 triple quadrupole (ABSciex) operating in positive electrospray ionization mode. The chromatographic separation was performed with the use of an Ascentis Express C18 2.7 μm 150 × 2.1 mm i.d. column protected with an Ascentis Express Cartridge Guard Column (Supelco Analytical with an Exp Titanium Hybrid Ferrule (Optimize technologies Inc.). The mobile phase consisted of A (water, 0.1% formic acid) and B (methanol, 0.1% formic acid) solutions. The following conditions were employed for chromatography: for unmodified nucleosides—0.13 mL/min flow, starting at 10% B for 0.1 min, ramping to 60% B over 2.4 min and re-equilibrating with 10% B for 4.5 min; for 5-oh(dC)—0.14 mL/min flow, starting at 5% B for 0.1 min, ramping to 70% B over 2.7 min and re-equilibrating with 5% B for 5.2 min; for dC modifications, and 8-oxo(dG)—0.14 mL/min flow, starting at 5% B for 0.5 min, ramping to 45% B over 8 min and re-equilibrating with5% B for 5.5 min. For mass spectrometry detection the multiple reaction monitoring (MRM) was implemented using the following mass transitions: 252.2/136.1 (dA), 228.2/112.1 (dC), 26,832/152.1 (dG), 243.2/127.0 (dT), 244.1/128 [5-oh(dC)], 284.1/168.1 [8-oxo(dG)].

### Analysis of mRNA expression level

Total RNA (1 μg) was reversely transcribed into cDNA using the High-Capacity cDNA Reverse Transcription Kit (Applied Biosystems). Relative expression levels were calculated using the comparative ΔCT method and related to the housekeeping gene β-actin. Primer sequences are listed in Table 1 below.

**Table 1:** Primer sequences for mRNA expression analyses

Target	Forward	Reverse
*Dcx*	TACCTGGGATTTTCCTTTGG	CTCGTTCGTCAAAATGTCCA
*Tuj-1*	CCAAGACAAGCAGCATCTGT	CAGAGCCAAGTGGACTCACA
*NeuN*	GAGTCTATGCCGCTGCTGAT	TTGCTAGTAGGGGGTGAAGC
*Gfap*	TCCTGGAACAGCAAAACAAG	CAGCCTCAGGTTGGTTTCAT
*β-actin*	CTTGATAGTTCGCCATGGAT	GGTCACTTACCTGGTGCCTA
*Cdh6*	GGAGCCGTAACCTTCCCATC	CGGTCTTCTGCTCTGTGCTT
*Nkd1*	TTGGGGAAAAGAGGCTGCTGA	CTCTAGAAGTGTCACCCACGAG

### Behavioral test

We employed the Novel Object Location (NOL) recognition task to evaluate the spatial pattern separation performance in mice. Animals were handled in 5-min sessions following a 3-day habituation period. For the short-term memory paradigm, the NOL task was performed in 2 sessions (1 training session and 1 test session) with intertrial intervals of 5 min. During the training session, animals were presented to 3 identical objects in a circular arena (46 cm diameter) equally distant from each other and the wall of the arena. Spatial cues were placed on the wall immediately behind the objects and animals were allowed to explore the objects for 10 min. In the test session, one of the objects was moved 40˚ away from its original position and we recorded the time animals spent exploring each object also during a total time of 10 min. Data acquisition was performed using the EthoVision live video tracking system (Noldus Information Technology Inc., VA, USA), and preference indexes for *Neil3*^*−/−*^ and control animals were determined by calculating the amount of time spent in the object at a new location divided by the sum of the total amount of time spent in each object. Results were plotted as mean ± SEM. and statistical analysis was performed applying unpaired Student’s t-test. For the long-term memory paradigm, the NOL task was performed in 4 sessions (3 training sessions and 1 test session) with intertrial intervals of 24 h. During the training sessions, animals were presented to 3 identical objects in a white square arena (50 cm × 50 cm), with a black cue card placed on the wall, equally distant to each other in a circular manner with the objects being spread apart 120°. The animals were allowed to explore the objects and the arena for 10 min. In the test session, one of the objects was moved 60° away from its original position and we recorded the animals exploring the objects and arena for 10 min. Data acquisition was performed using the ANY-Maze video tracking tool (Stoelting) and preference indexes for wildtype and *Neil3*^*−/−*^ animals were determined by calculating the amount of time investigating the novel zone divided by the sum of the total amount of time investigating all objects. Results were plotted in GraphPad Prism (Dotmatics) as mean ± SEM and statistical analysis was performed using unpaired Student's t-test.

### BrdU injection

BrdU injections were administrated to animals undergoing the long-term memory paradigm. BrdU (Sigma, 100 mg/kg body weight) was delivered to mice intraperitoneally every 24 h for 7 days, including three injections prior to and four injections during the behavioral task. Animals were sacrificed 24 h after the final test session.

### Frozen section immunohistochemistry

Mice were transcardially perfused with saline solution (BBraun) before brain removal. Brains were fixed in 4% PFA for at least 48 h, then frozen sectioned into 30um sagittal sections. DNA hydrolysis was performed using 1 M HCl for 1 h at 37 °C, the reaction was neutralized by incubating in 0.1 M Sodium Borate (pH 8.5) for 10 min at RT and sections were washed thoroughly with PBS. Antigen retrieval was performed using sodium citrate 40 mM, pH 6.0, incubated at 99 °C for 4 min. Subsequently, sections were blocked with 5% BSA 5% NGS and 0.1% Triton-X in PBS for 1 h. After blocking, sections were incubated with primary antibodies in the antibody dilution buffer (1% BSA 1% NGS, and 0.1% Triton-X in PBS) overnight at 4 °C. After washing in PBS with 0.1% Tween-20, sections were incubated with secondary antibodies for 2 h at RT (protected from light). Samples were mounted in Superfrost™ Plus Adhesion Microscope Slides (Epredia) while submerged and let to dry overnight. Samples were then incubated with DAPI staining solution (1ug/ml, Thermo Scientific) for 15 min before being cover slipped with ProLong™ Gold Antifade with DAPI Mountant (Invitrogen).

### Antibodies

Primary antibodies used were anti-Ki67 (rat IgG2a, 1:500, ThermoFisher, Cat. No. 14–5698-82, RRID: AB_10854564), anti-BrdU (mouse IgG2a, 1:500, Invitrogen, Cat. No. MA3-071, RRID: AB_ 10,986,341), anti-DCX (rabbit IgGs, 1:1000, Cell Signaling, Cat. No. 4604S, RRID: AB_ 561,007), anti-SOX2 (rabbit IgGs, 1:1000, Sigma-Aldrich, Cat. No. PA1-094, RRID: AB_ 2,539,862), anti-NeuroD1 (rabbit IgGs, 1:500, Abcam, Cat. No. ab213725, RRID: AB_2801303), anti-PROX1 (rabbit, 1:1000, Porteintech, Cat. No. 11067–2-AP, RRID: AB_2268804), anti-Nestin (mouse IgGs, 1:200, Millipore Cat. No. MAB5326, RRID:AB_2251134), rabbit anti-GFAP (rabbit IgGs, 1:500, Agilent Cat. NO. Z0334, RRID:AB_10013382), and anti-NeuN (mouse IgG1, 1:500, Millipore, Cat.No. MAB377, RRID: AB_2298772). Secondary antibodies were Alexa Fluor® 488 anti-mouse IgG1 (1:1000, Cat. No. A-21121, RRID: 2,535,764), Alexa Fluor® 488 anti-mouse IgG2a (1:1000, Cat. No. A-21131, RRID: AB_2535771), Alexa Fluor® 488 anti-rabbit (1:1000, Cat. No. A32731, RRID: AB_2633280), Alexa Fluor® 555 anti-mouse IgG1 (1:1000, Cat. No. A-31570, RRID: AB_2536180), Alexa Fluor® 555 anti-rabbit (1:1000, Cat. No. A-31572, RRID: AB_162543), Alexa Fluor® 647 anti-mouse IgG2a (1:1000, Cat. No. A-21241, RRID: AB_2535810), Alexa Fluor® 647 anti-rabbit (1:1000, Cat. No. A-21244, RRID: AB_2535812) Alexa Fluor® 647 anti-rat (1:1000, Cat. No. A-21247, RRID: AB_141778) and Alexa Fluor® 647 anti-guinea pig (1:1000, Cat. No. A-21450, RRID: AB_141882).

### 3D-image analysis

Microscopy was carried out using a Zeiss LSM 880 confocal laser scanning microscope equipped with a 40X oil immersion objective. 3D-image analysis was performed using IMARIS 9 (Oxford Instruments). The surface tool was utilized to select the granular and subgranular zones in the DG, allowing for calculating their volume across all visible stacks. For cell density analysis, the Cell Counter plugin in FIJI [38] was used to manually count the number of Ki67^+^, SOX2^+^, DCX^+^, BrdU^+^, and NeuroD1^+^ cells in their respective channels. For fluorescent voxel analysis, the initial surface created in IMARIS was masked in the BrdU channel. A new surface was then generated by selecting only BrdU-positive cells within this mask, and the fluorescent voxel counts were obtained from this surface for NeuroD1 or Prox1 in their corresponding channels. Results were calculated by dividing the number of positive cells or the number of voxels by the volume in mm^3^. Data were plotted as mean ± SEM using GraphPad (Dotmatics), and statistical analysis was performed using two-way ANOVA with Tukey’s multiple comparison test.

### RNAseq data and analysis

The RNAseq dataset consisted of 6 samples, including 3 × wildtype samples (3 m-DG) and 3 × *Neil3*^*−/−*^ samples (3 m-DG). It is a part of the RNAseq dataset (GSE175360) [24] and is available in the Gene Expression Omnibus repository (GEO). Sample and sequencing details were described in Bugaj et al., 2024. Briefly, the raw data of gene expression was obtained from BGI and the normalized expression levels were transformed by the variance stabilizing function DESeq2::vst. DESeq2 was used to test for differential expression between genotypes in adult DG. Differential gene expression was determined by the threshold of adjusted p-value < 0.05 and ABS(log2 fold change) > 0.6. For the PANTHER pathway over-representation analysis, the list of DEGs was uploaded to the online version of PANTHER Classification System using Binomial test and Bonferroni correction and mouse genome as background.

### Cre-dependent EYFP expression in DCX-expressing hippocampal newborn neurons

*Neil3*^+/-^ ::DCX-CreER2 (control) and *Neil3*^-/-^::DCX-CreER2 mice (mean age of 2.5 months) were injected with 1ul of AAV2/9-dio-eYFP (Addgene Plasmid #27,056, obtained from the University of Pennsylvania vector core) at AP-3.5 mm, ML-3 mm, and DV-4 mm to induce Cre-dependent EYFP expression in hippocampal newborn neurons. DCX-CreER2 mice carry a tamoxifen-inducible CreERT2 and tamoxifen (20 µg/g body weight) was administered three weeks post-injection. One week later (4 weeks of viral expression), the animals were euthanized via rapid decapitation under ketamine (80 mg/kg) anesthesia.

### Patch-clamp Recording

Brains were removed after decapitation and placed in ice-cold artificial cerebrospinal fluid (ACSF)/sucrose solution (in mM: KCl, 2.49; NaH_2_PO_4_, NaHCO_3_, 26; glucose, 10; sucrose, 252; CaCl_2_, 1; MgCl_2_, 4) (leao 2012). Horizontal slices (400 μm) were collected on a vibratome (VT1200, Leica) and transferred to a chamber filled with recording ACSF (in mM: NaCl, 124; KCl, 3.5; NaH_2_PO_4_, 1.25; MgCl_2_, 1.5; CaCl_2_, 1.5; NaHCO_3_, 30; glucose, 10), constantly bubbled with 95% O_2_ and 5% CO_2_ (White-Martins). For whole cell patch clamp recordings, slices were transferred to the stage of an upright microscope (Zeiss). Recording pipettes were filled either with a K-gluconate-based solution (in mm: 17.5 KCl, 122.5 K-gluconate, 9 NaCl, 1 MgCl_2_, 3 Mg-ATP, 0.3 GTP-Tris, 1 HEPES, 0.2 EGTA; pH was adjusted to 7.2 using KOH) supplemented with 1–2% biocytin (Sigma-Aldrich). Granule cells were patched under visual guidance using an upright microscope equipped with differential interference contrast optics (Leao 2009 kv7/kcnq). After obtaining whole-cell configuration, current-clamp recordings were obtained to analyze the firing properties of DG cells using an Axopatch 200B amplifier.

## Supplementary Information

Below is the link to the electronic supplementary material.

Supplementary file1 (PDF 291 KB)

## Data Availability

The RNAseq data is a part of the RNAseq dataset (GSE175360) [24] and is available in the Gene Expression Omnibus repository (GEO). Other data generated and analyzed during the current study are available from the corresponding author upon request.
