# Supplementary material for: NEIL3 influences adult neurogenesis and behavioral pattern separation via WNT signaling
Source: Cell Mol Life Sci. 2025 Mar 4;82(1):101. doi: 10.1007/s00018-025-05629-5 (PMC11880487; doi:10.1007/s00018-025-05629-5)
Supplement: Supplementary file 1 — Supplementary file1 (PDF 291 KB) [file 18_2025_5629_MOESM1_ESM.pdf]

Supplemental Figures

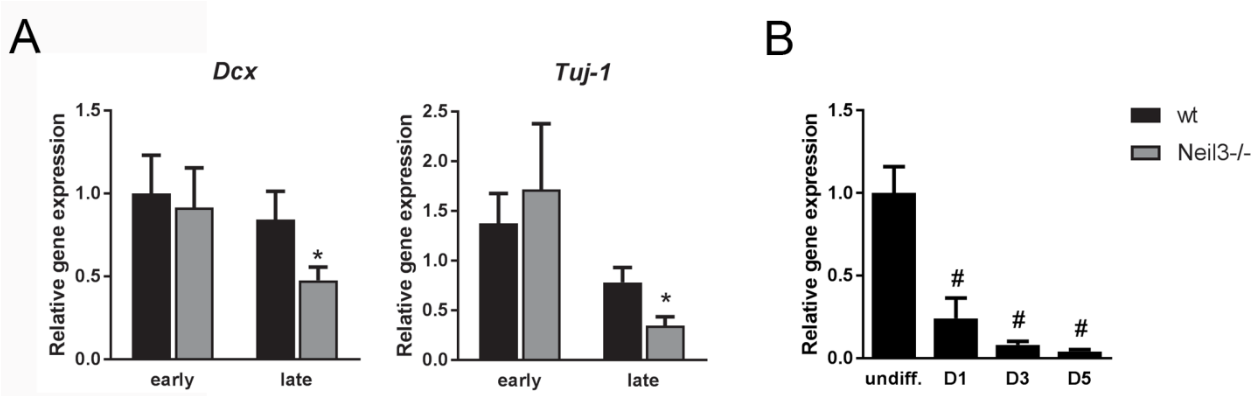

Figure S1. Neil3 is expressed in proliferating NSPCs and promotes survival of neuronal lineage cells. NSPCs were cultured from postnatal day 5 wildtype (wt) and *Neil3*$^{-/-}$ hippocampus. A) Gene expression analysis of neuronal marker at passage 2-5 (early) and passage 6-17 (late). B) Gene expression analysis of *Neil3* in undifferentiated (undiff.) and differentiated NSPCs (p5-p11) for one day (D1), three days (D3) and five days (D5). Statistical analyses were done in GraphPad Prism Software v10.1 using 2-way ANOVA with Tukey's multiple comparisons test in (A) or one-way ANOVA in (B). Data are presented as mean ± SEM from NSPC preparation of at least three mice each genotype with $^{*}p < 0.05$ as compared to wt and $^{\#}p < 0.05$ as compared to undiff. wt.

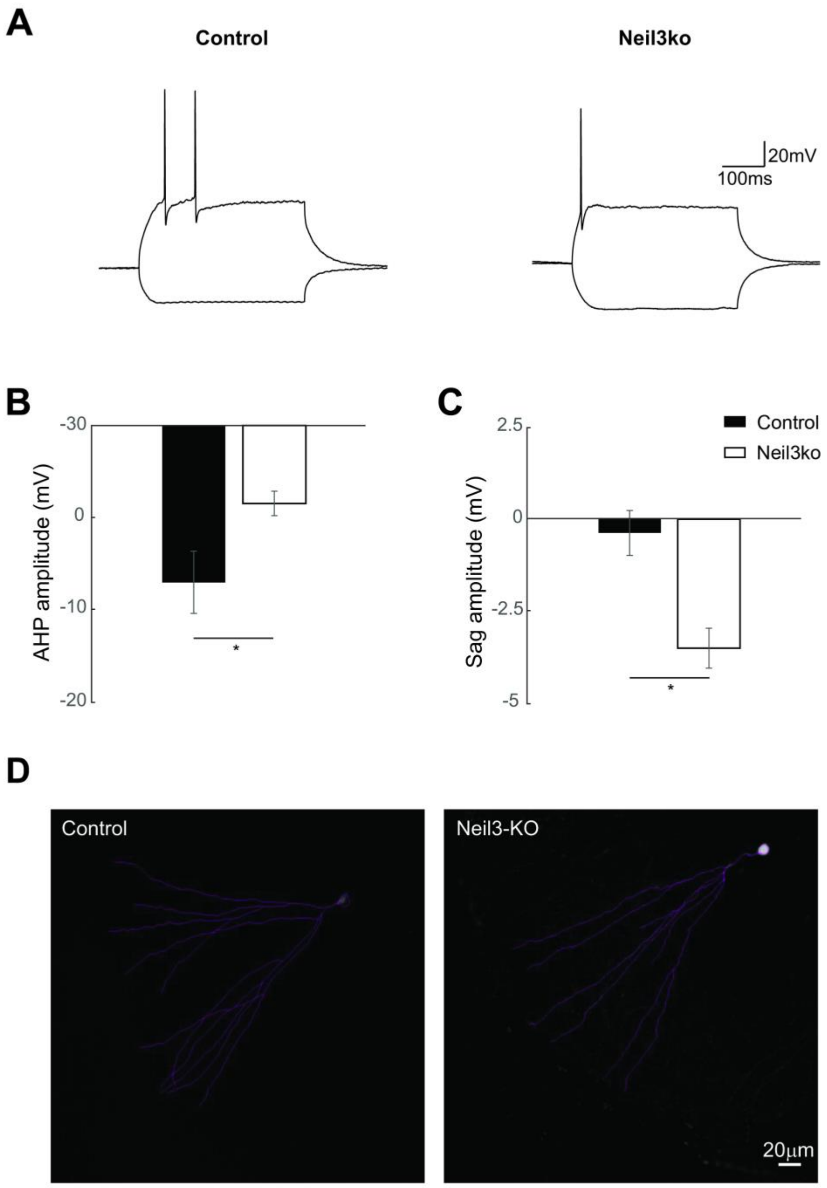

Figure S2. Membrane properties of adult granule cells from *Neil3*$^{-/-}$ mice. A) Example of current clamp recordings of DG neurons in *Neil3*$^{-/+}$ (control) and *Neil3*$^{-/-}$ (Neil3ko) mice in response to a 400 ms long current step (-100pA and 100 pA). B) Mean amplitude of the afterhyperpolarization potential. Data are presented as mean ± SEM (n=24 cells), *p<0.05. C) Sag amplitude following a hyperpolarizing current step. Data are presented as mean ± SEM (n=24 cells), *p<0.05. D) Biocytin-filled adult granule cells.

Supplementary experimental procedures

Sholl analysis

To analyze dendritic morphology, slices were transferred from the recording chamber to a container with 4% paraformaldehyde in PBS. Slices were fixed overnight or longer. After washing in PBS for 10 minutes, slices were incubated overnight in 0.1% streptavidin conjugated with the fluorochrome Alexa 488 (Life Sciences). Slices were then washed and mounted with mowiol. Photomicrographs of granule cells were obtained in a laser scanning confocal microscope or a conventional microscope equipped with epifluorescence (Zeiss). Dendrites were traced using the ImageJ plugin NeuronJ (Leao et al., 2012). NeuronJ files were them imported into Matlab for automated Sholl analysis using the 'Bonfire' program (Langhammer et al., 2010).

References

Langhammer, C. G., M. L. Previtera, E. S. Sweet, S. S. Sran, M. Chen and B. L. Firestein (2010). "Automated Sholl analysis of digitized neuronal morphology at multiple scales: Whole cell Sholl analysis versus Sholl analysis of arbor subregions." Cytometry A 77(12): 1160-1168.

Leao, R. N., S. Mikulovic, K. E. Leao, H. Munguba, H. Gezelius, A. Enjin, K. Patra, A. Eriksson, L. M. Loew, A. B. Tort, et al. (2012). "OLM interneurons differentially modulate CA3 and entorhinal inputs to hippocampal CA1 neurons." Nat Neurosci 15(11): 1524-1530.
